# Challenges and considerations for whole-genome-based antimicrobial resistance plasmid investigations

**DOI:** 10.1128/aac.01097-25

**Published:** 2025-11-11

**Authors:** Jia Qi Beh, Ryan R. Wick, Benjamin P. Howden, Christopher H. Connor, Jessica R. Webb

**Affiliations:** 1 Department of Microbiology and Immunology at the Peter Doherty Institute for Infection and Immunity, The University of Melbourne, Melbourne, Australia; 2 Centre for Pathogen Genomics, University of Melbourne, Parkville, Victoria, Australia; 3 Microbiological Diagnostic Unit Public Health Laboratory, Department of Microbiology and Immunology at the Peter Doherty Institute for Infection & Immunity, The University of Melbourne, Melbourne, Victoria, Australia; 4 Department of Infectious Diseases & Immunology, Austin Health 740438 , Heidelberg, Victoria, Australia; 5 School of Biological Sciences, The University of Adelaide 110455, Adelaide, South Australia, Australia; Samsung Medical Center, Seoul, Republic of Korea

**Keywords:** plasmid, antimicrobial resistance, bacteria, public health, genomics

## Abstract

Plasmids are self-replicating, extrachromosomal genetic elements that serve as vehicles for antimicrobial resistance (AMR) genes. In bacteria, plasmids frequently carry critical AMR genes such as extended spectrum beta-lactamases (*bla_CTX-M_*) in gram-negative and glycopeptide resistance gene (*vanA*) in gram-positive species. Plasmid sequences are genetically diverse within and across taxa, with the PLSDB database recording up to 72,360 non-redundant sequences in May 2024. Horizontal transfer of plasmids continues to threaten the effectiveness of last-resort antibiotics, especially as plasmids can disseminate horizontally crossing taxonomic boundaries. Whole-genome sequencing is a powerful approach for investigating AMR plasmids, yet there are several challenges facing bioinformatic tools and databases. Here, we review those challenges and their implications for AMR plasmid research as well as summarizing key bioinformatic analyses and tools used in AMR plasmid investigations. The review highlights how genomics has revolutionized AMR plasmid studies in drug-resistant pathogens and provides insights on the current limitations and future challenges to leverage plasmid genomics in public health research.

## INTRODUCTION

Antimicrobial resistance (AMR) is a major concern in public health, with over 5 million deaths associated with bacterial AMR (1). AMR can be conferred by *de novo* mutations on the chromosome or through the acquisition of genes which interfere with the mechanism of action of antibiotics—also known as antimicrobial resistance genes (ARGs). Bacterial ARGs can be chromosomal or carried on plasmids. Plasmids are extrachromosomal genetic elements that replicate independently of their host chromosome. These small, often circular elements are frequent “vectors” of ARGs, allowing the horizontal dissemination of resistance genes between bacteria (2, 3).

Plasmids can evolve using the same genetic changes as chromosomal DNA, such as nucleotide changes, gene gain and loss, structural changes (large indels), and recombination. However, the ability of plasmids to transfer horizontally between bacteria greatly complicates reconstruction of their “true” evolutionary history. One approach is to look at accumulated mutations in the core or “backbone” regions of plasmids from the same lineage (4); however, this ignores any structural variation and is not informative of horizontal transfer history. In addition, plasmids frequently exchange genetic material with the chromosome and with each other, often resulting in vanishingly small plasmid backbones to examine. Frequent recombination also permits plasmids to evolve in ways that the chromosome cannot, such as forming plasmid co-integrates (where two distinct plasmids merge together) or integrating into the chromosome (5, 6). Different plasmids evolve at different rates and in different bacterial hosts following conjugation (7). Different regions (“core” and “accessory”) of the plasmids may also evolve at different rates. Environmental conditions also contribute to how fast plasmids within a population evolve, adapt, and spread between members (8, 9). The varied ways in which plasmids move, exchange DNA, and evolve pose challenges for bioinformatic detection of sequences and consistent classification.

Laboratory-based methods to characterize and type AMR plasmids can be laborious and hinder real-time plasmid transmission detection in clinical settings, which can be crucial in outbreak investigations. *In silico* plasmid analysis has the potential to replace laboratory methods, but important challenges remain on key areas, including constructing complete plasmid replicons from short reads, identifying and typing novel and linear plasmids, and sampling and sequencing biases in the plasmid sequence databases.

## SIGNIFICANCE OF PLASMIDS CARRYING AMR GENES

Bacterial pathogens can sometimes have a considerable portion (more than a quarter) of their genome made up of accessory elements, including plasmids (10), contributing to their exceptional ability to adapt in environments with high antibiotic selection pressure. Although plasmids play a large role in pathogen adaptation and survival, significant knowledge gaps exist within the niche of plasmid genomics. These challenges can be attributed to the dynamic nature of plasmids and the quality of genome assemblies.

Plasmid recombination plays an important role in the spread of AMR genes in bacteria. Some plasmids undergo recombination with the chromosome or other plasmids in the same host or in different hosts following conjugation (4). Recombination has been shown to drive the evolution of certain Inc plasmid groups in gram-negative species (11–13). This process is mediated by transposable elements (TEs) that can excise and form circular intermediates and incorporate into a different location in the host genome. Notable examples of TE which facilitate transposition and integration of AMR genes into plasmids are the insertion sequence (IS) families IS*26*-like (8) and IS*91* (9) in gram-negative bacteria. The transposition of AMR genes between plasmids was further demonstrated via the nested, Russian doll-like model observed in the Tn*4401* transposon, which drives carbapenem resistance dissemination in Enterobacteriaceae (14), and in the dissemination of *bla_CTX-M-15_* in strains with extended-spectrum β-lactamases (ESBLs) (4). Additionally, co-integration of multiple plasmids or plasmid segments may result in plasmids carrying multiple AMR determinants (15–19). This plastic nature presents challenges for studying AMR plasmid evolution and reconstructing the AMR gene movements within a complex bacterial community, such as the human microbiome (20).

In this review, we discuss the challenges and considerations of using whole-genome sequencing (WGS) to study AMR plasmids. We summarize the different *in silico* analyses and tools used in AMR plasmid identification, typing, annotation, and transmission analysis in bacteria using short- and/or long-read sequences as input.

## USING WHOLE-GENOME SEQUENCING TO STUDY AMR PLASMIDS

Traditionally, plasmids were distinguished and physically separated from the bacterial chromosome based on size or structure, using PCR primers targeting specific plasmid replicase genes (21–23). WGS and bioinformatic tools now seek to replace experimental laboratory methods to predict and reconstruct plasmids based on sequence similarity. Sequencing offers several advantages over traditional gel-based methods. First, sequencing will ideally capture all plasmids present in a single pure colony (24, 25). Second, sequencing data can be used to identify recombination events between highly similar plasmid sequences. This is particularly useful for outbreak investigations and for studying plasmid sharing across One Health sectors (26–28). Third, WGS allows the study of the genetic context (location, association with TE, etc.) of a particular gene of interest to be determined. This is particularly useful for understanding the transmission and evolutionary history of critical genes (29–31). Finally, WGS enables large-scale study of bacterial plasmids, which can be too laborious using laboratory-based methods (32).

Bacterial WGS studies involve sequencing whole-genome DNA on short- or long-read platforms, followed by assembly into contigs. In short-read sequencing (e.g., Illumina) (33, 34), the extracted bacterial DNA is first fragmented, and adapters are ligated onto the fragments to create DNA libraries. The DNA libraries then undergo bridge amplification on the flow cell to generate clusters of DNA fragments. A complementary strand of DNA is synthesized using the original strand as the template, and the process is repeated many times to generate clonal clusters of the same DNA fragment. As new bases are incorporated onto the complementary strands, the fluorescent signals emitted from each cluster are captured by the sequencer and converted into corresponding DNA bases. The reads generated in Illumina are typically around 150 bp, depending on the sequencing instrument. Illumina reads are assembled into contigs, which together make up a “draft” genome or assembly. Short-read assemblies are nearly always fragmented due to the presence of repetitive genomic elements, including transposable elements that carry AMR genes (35). Thus, studying the genetic context of AMR genes, whether plasmid or chromosomally encoded, using only short reads can be challenging. Short-read assemblies can resolve plasmids without repeats longer than the insert size of the sequencing library, which is usually the case for small plasmids (36). However, AMR plasmids are typically larger and contain repeats, making them difficult to assemble with short reads (37).

Long-read sequencing platforms from Oxford Nanopore Technologies (ONT) and Pacific Biosciences (PacBio) generate longer reads, which usually allow for complete genome assembly (38). However, the per-base cost of long-read sequencing is on average higher than that of short-read sequencing (39). Long-read sequencing can also perform poorly for small (<20 kbp) plasmids due to biases in DNA preparation and algorithmic challenges in assembly tools (38, 40, 41). Earlier long-read platforms were limited by lower basecalling accuracy, especially in homopolymers, but recent improvements now allow for accurate assemblies without short-read polishing (42, 43). Thus, long-read sequencing is typically used to generate complete replicons to understand the AMR gene context and to construct plasmid references (e.g., index plasmids) for sequence comparison, while short-read sequencing is more cost-effective and better suited to recovering small plasmids (41). Hybrid assembly, where both long and short reads are combined to generate assemblies, offers the advantages of both technologies and was recognized as the “gold” standard for constructing reference or “index” plasmids for alignment comparison (38). Due to improvements in basecalling accuracy, long-read-only assemblies are now also suitable, though care must be taken to ensure small plasmids are not missed (44, 45).

## *IN SILICO* ANALYSES FOR AMR PLASMIDS IN BACTERIA

**Fig 1:**
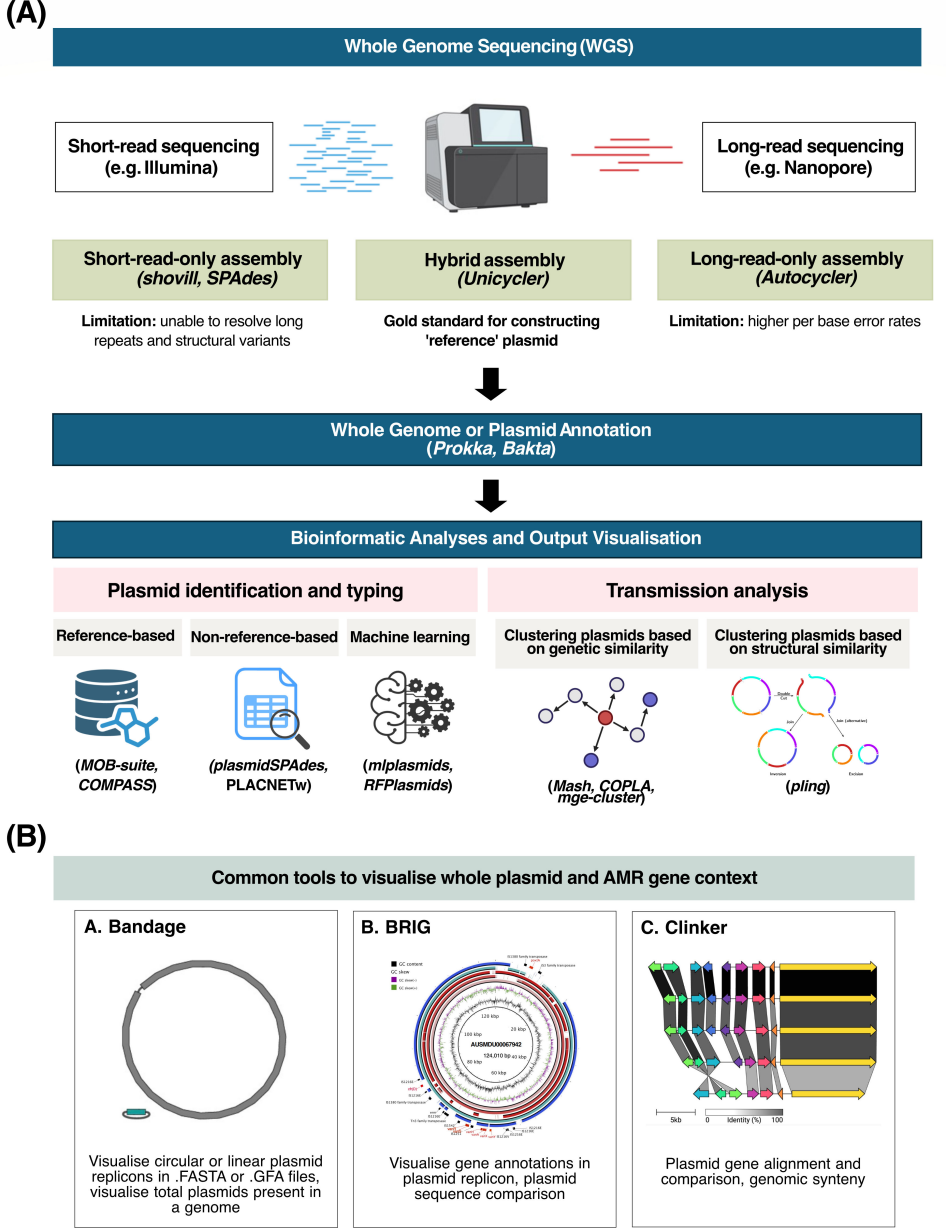
*In silico* analyses to study antimicrobial resistance (AMR) plasmids in bacteria. (**A**) The initial step involves genome assembly to reconstruct a draft (short-read-only) or complete (long-read) bacterial assembly. The genome assembly (collection of contigs) can be annotated with Prokka or Bakta (all open reading frames [ORFs]) or other specific gene detection tools such as Abricate (for resistance genes) and ISfinder (for insertion sequences). Annotated genomes can then be used for downstream analyses. Plasmid prediction and typing are often run simultaneously, and prediction accuracy varies depending on the assembly completeness level (draft or complete). Transmission analysis involves reconstructing the route of transmission of an AMR plasmid by sequence comparison to an index plasmid using a pre-established or local SNP threshold. (**B**) To visualize whole plasmids or specific regions of interest (e.g., flanking regions of an AMR gene), various tools can be used such as Bandage (46), BRIG (47, 48), or Clinker (49).

Here, we summarize the key bioinformatic analyses for studying AMR plasmids in bacteria (Fig. 1). To reconstruct plasmid sequences, bacterial short and/or long reads need to be first assembled into contigs. Quality control (QC) checks are performed on the assembled contigs to ensure that poor quality genomes (e.g., genomes that are too fragmented) are excluded from further analyses. QC can be performed by checking the number of contigs generated, the median contig length (N50) for the entire genome, or in cases of short-read assemblies by counting the number of dead ends in the assembly graph. Genome assemblies that passed QC checks can then be analyzed for various purposes. We outline several key analyses below, including plasmid reconstruction and typing, plasmid annotation, and transmission analysis. Many analyses can be performed on either draft assembly from short reads or complete plasmid sequences from long-read or hybrid assembly. Some analyses (e.g., plasmid transmission) require a plasmid “reference,” which can be obtained from public repositories (NCBI or PLSDB) or generated locally using long-read sequencing.

### Identifying and typing plasmids

The first step and possibly most challenging step in short-read bacterial AMR plasmid studies is identifying plasmid sequences from WGS data. Plasmids are abundant with mobile genetic elements, such as transposons and IS elements. These elements create repeats in the DNA sequence, which short-read genome assembly methods cannot accurately resolve. IS elements also result in genome assemblies with a high number of contigs, as the algorithms for piecing together short read data cannot distinguish where these repeats belong. This leads to fragmented genomic segments, including plasmid sequences. Further discrimination between contigs that belong to plasmids and those belonging to the chromosome is challenged by transposable elements, which can co-occur simultaneously on the chromosome and plasmid.

Despite these challenges, several bioinformatic tools have been developed to distinguish plasmid sequences from genomic data (Table 1). Here, we focus on the tools for reconstructing or predicting plasmid sequences from short-read assemblies, as identifying plasmids in long-read assemblies is often more direct, with complete plasmid assembly usually possible, although additional steps are still needed to confirm plasmid identity. Many of these tools were used simultaneously to type plasmids, as the presence of a plasmid family or lineage-specific genes is equivalent to plasmid presence. Popular examples include MOB-suite (50, 51) and PlasmidFinder (52), with more recent tools (e.g., mlplasmids [53]) incorporating machine learning (ML) algorithms for making predictions. When it comes to choosing a tool, there is often a compromise between specificity, sensitivity, accuracy, precision, and usability (command line versus graphical interface). For instance, typing tools that rely on an existing plasmid sequence database, such as MOB-typer and COMPASS, have higher specificity, as these tools can detect highly similar plasmids—but at the same time could miss out on novel plasmid sequences leading to reduced sensitivity (50, 54). ML tools have higher precision (higher rates of detecting putative plasmid contigs), but performance is heavily dependent on the input training data set and model used.

**TABLE 1:** Tools for plasmid prediction and typing published between 2014 and 2024^*a*^

Tools	Publication year	Description	Limitations
Reference-based			
Plasmid Finder (52)	2014	Plasmid prediction and typing from bacterial draft genomes. Detects homologous plasmid sequences based on replicon typing using the BLASTn algorithm (52). Originally established and curated for *Enterobacteriaceae* plasmids. Users can run BLAST using their own sequence to look for similar plasmids in the database. Available as a web tool and has been incorporated into various bioinformatic pipelines, such as ARIBA, TORMES, Bactopia, BacSeq, and staramr. Database was last updated on 18 January 2023. Accessible at: https://cge.food.dtu.dk/services/PlasmidFinder/	Reference-based tools identify only known plasmid sequences, and performance is highly dependent on the robustness of the underlying database. Novel plasmids or plasmids from underrepresented species are often undetected. These methods also often fail to capture structural variations in plasmids.
MOB-suite (50, 51)	2018	A multi-utility tool for plasmid detection (mob-recon), typing (mob-typer), and clustering (mob-cluster) from draft or complete assemblies. Plasmid typing is performed based on plasmid replicon family, relaxase, mate-pair formation, and transferability, before clustering plasmids based on pairwise Mash distance against a reference plasmid database. MOB-suite demonstrates comparable specificity and higher sensitivity than Plasmid Finder. Lower tendency of merging similar plasmids than plasmidSPAdes. Available as a command line tool only. Database was last updated on 4 June 2024. Accessible at: https://github.com/phac-nml/mob-suite	
COMPASS (54)	2020	A comprehensive plasmid database (*N* = 12,084) including metadata from over 1,500 species worldwide spanning considerable time span, with reduced redundant plasmid sequences compared to MOB-suite, pATLAS, and PLSDB. Offers *in silico* plasmid typing based on replicon and mobilization (MOB) systems. Database was curated from NCBI Nucleotide (as of 17 June 2018). Accessible at: https://github.com/itsmeludo/COMPASS	
PlasmidSeeker (55)	2018	Plasmid assembly and detection using short-read sequencing data. Uses *k*-mer abundance to distinguish between plasmid and chromosomal contigs. Users have the option to build their own plasmid database. Database was last updated on November 2020. Accessible at: https://github.com/bioinfo-ut/PlasmidSeeker	
Non-reference based			
plasmidSPAdes (56)	2016	Plasmid assembly and prediction using short-read sequence data. plasmidSPAdes runs on a reference-free approach to distinguish between “plasmid” and “chromosomal” sequences based on the median read coverage from the assembly graph. Accessible at: https://github.com/ablab/spades	Non-reference-based tools rely on read coverage or assembly graphs to distinguish plasmid sequences from chromosomal sequences. PlasmidSPAdes has a higher chance of missing out small plasmids or misinterpreting short chromosomal sequences as plasmids, as it struggles to resolve tandem repeat regions in the assembly graphs. Additionally, plasmidSPAdes does not perform well when the plasmid sequencing coverage is similar to the chromosome (low copy number plasmids). PLACNETs accepts only short reads as input, and assembly can be impacted by the presence of repetitive elements in the plasmid sequence.
PLACNETw (57)	2017	Web tool designed based on PLACNET, a graph-based tool to identify and reconstruct plasmid contigs. Performs plasmid assembly and prediction using paired-end reads. Graphical user interface available for users to inspect plasmid network with nodes showing plasmid-specific genes. Accessible at: https://castillo.dicom.unican.es/upload/	
Tools that use machine learning (ML) algorithms			
mlplasmids (53)	2018	Uses the support vector machine (SVM) models as a machine learning classifier and pentamer frequencies to classify short-read contigs as plasmid or chromosome derived. Benchmarked using three species: *Enterococcus faecium*, *Klebsiella pneumoniae,* and *Escherichia coli*. Available as an R package or web server. Accessible at: https://gitlab.com/sirarredondo/analysis_mlplasmids (R package) or https://sarredondo.shinyapps.io/mlplasmids (Web)	Machine learning (ML) based tools rely on an “input” training data set for plasmid prediction. The performance of these tools depends heavily on the quality and size of the training data set, the quality of the input assemblies, and the training model used.
RFPlasmid (58)	2021	Plasmid prediction using random forest models from short-read assemblies and metagenomics data. Plasmid prediction using a combination of features including *k*-mer composition and plasmid protein and replicon sequences, contig lengths, and gene count. Supports 17 different bacterial taxa (more diverse than mlplasmids) and taxon-agnostic organisms. Available as a command line tool. Accessible at: https://github.com/aldertzomer/RFPlasmid	
SourceFinder (59)	2022	Machine learning tool using random forest classifier and *k*-mer frequencies to predict plasmid and bacteriophage sequences from draft or complete assemblies. Available as CGE Server web tool. Accessible at: https://cge.food.dtu.dk/services/SourceFinder/	
Deeplasmid (60)	2022	Deep learning algorithm to distinguish plasmid from chromosomal sequences using assembly or metagenomics data. Prediction based on multiple parameters, such as sequence composition (GC and homopolymer), plasmid-specific genes and protein domains, and gene density within the contig. Uses a shorter window length (300 bp) compared to other prediction tools. Available as a command line tool. Accessible at: https://github.com/wandreopoulos/deeplasmid	
Plasmer (61)	2023	Machine learning tool that uses shared *k*-mer and genomic features (alignment and replicon distribution scores) for plasmid prediction from assemblies. Demonstrated excellent sensitivity and specificity on contigs > 500 bp. Available as a command line tool. Accessible at: https://github.com/nekokoe/Plasmer	
geNomad (62)	2023	A classification and annotation framework that uses gene content and deep neural network to identify plasmid and viruses. The tool uses a hybrid approach that combines alignment-free and gene-based models for prediction. Available as a command line tool. Accessible at: https://github.com/apcamargo/genomad	
PlasmidHunter (63)	2024	Plasmid prediction tool using machine learning based on gene content profile. Accepts assemblies and metagenomes as input. Higher precision for larger plasmids (10 kbp versus 5 kbp). Available as command line tool. Accessible at: https://github.com/tianrenmaogithub/PlasmidHunter	

^*a*^ Plasmid prediction tools can be classified as reference-based, non-reference-based, and those dependent on machine learning (ML) algorithms.

Various tools are available to perform plasmid typing on either draft (short-read) or complete (long-read or hybrid) assemblies. Compared to laboratory-based typing methods using plasmid-specific primers and PCR, *in silico* plasmid typing tools offer more flexibility and a universal plasmid sequence database for interlaboratory comparison. Plasmid typing can be performed with or without a reference database. Reference-based approach relies on an existing plasmid sequence database, which can be manually (e.g., PlasmidSeeker [55], Kraken classifier [64]) or publicly (e.g., PlasmidFinder [52], ARIBA [65], and bakta [66]) curated. These sequences can be retrieved from public repositories, such as NCBI (e.g., RefSeq for genes) or UniProt (e.g., UniRef, for proteins). Many of these typing tools rely on the conserved regions of plasmid replication initiation (Rep) and relaxase (Mob) genes for mobility, or mate pair formation (MPF typing)—the latter two restricted to conjugative and mobilizable plasmids (67). Rep, MOB, and MPF typing are largely restricted to the typing of known plasmid types and do not perform well with data sets that have considerably large numbers of novel or non-typeable plasmids, such as environmental samples (68). Typing tools that run on a reference-based approach accept reads or assemblies as input. When reads are provided, the reads are usually pre-assembled into contigs before running the prediction step. In contrast, non-reference-based approaches predict plasmids based on read depth or contig circularization, often requiring reads or assembly graphs as input. Plasmid prediction tools that incorporate ML algorithms vary in predictive ability and accuracy, as these tools rely heavily on the parameters used for prediction (gene content or *k*-mer composition) and the quality of the input assemblies. Moreover, the training data set is frequently biased toward Enterobacteriaceae, leading to poor performance on other species.

### Plasmid gene annotation and visualization

Plasmid genes can be predicted using genome annotation tools, such as Prokka (69) or Bakta (66). In the context of AMR plasmid investigation, ARGs are screened using specialized tools such as ABRicate, AMRFinderPlus (70), abritAMR (71), or the NCBI Pathogen Detection Project (72). These screening tools require a curated database of AMR genes, such as the Comprehensive Antibiotic Resistance Database (CARD) (73). ARG detection is useful for different purposes. First, it can be used to locate the genomic position of an ARG on a plasmid and identify if the same gene is located on the same plasmid across genetically dissimilar isolates, which could indicate plasmid transfer. This is useful as an early indication of a possible hospital outbreak and help inform control strategies (74, 75). Second, ARG detection indicates which ARGs could be co-located on the same plasmid. For example, co-localization of genes encoding resistance to last-line antimicrobials vancomycin (*vanA*) and linezolid (*cfr, cfr*(D)*, optrA,* and *poxtA*) on the same plasmid has been identified in *Enterococcus faecium* (47, 76). IS elements were frequently detected upstream and/or downstream of these ARGs, suggesting their role in mediating integration of ARGs into different plasmids (77–79). Another study found that a significant proportion (78%) of 2,591 ARG-encoding plasmids in Enterobacteriaceae comprised of multiple ARGs, with 84% of ARGs co-located on resistance islands (80). Together, these findings highlight the dynamic nature of AMR plasmids and the role of IS elements in mediating ARG plasmid recombination and spread across different pathogenic strains in bacteria.

To study if an ARG could have been disseminated through IS elements, the flanking regions (or genomic context) of the gene need to be characterized. Specialized tools such as ISfinder (81) can be used to screen for the presence of IS elements upstream and downstream of an ARG. To study the genomic context surrounding a plasmid AMR gene, the contig with the gene needs to be first identified and extracted from the genome assembly. This can be achieved using Bandage (46) for single genome, or contig-puller (https://github.com/kwongj/contig-puller) for multiple genomes. Immediate regions around the gene can also be extracted for alignment comparison using Flanker (82). Bandage (Fig. 1) is an open-source, desktop application for interactive genome visualization and accepts assembly graphs or FASTA sequences as input. Users have the option to run a local BLAST (given that BLAST is pre-installed) on plasmid contigs and check for plasmid conformation (e.g., circular or linear). Once the AMR contig has been identified, the contig sequence can be saved, annotated, and visualized. Often, contigs from short-read assemblies are short in length due to truncation by repetitive sequences from IS elements flanking the AMR genes, making it difficult to study the full context upstream and downstream of the gene. In this case, long-read sequencing is needed to close the gap between these repeat regions.

Various genome viewer platforms are available for visualizing AMR plasmids and genetic contexts (Fig. 1). The Integrative Genomics Viewer (IGV) (https://igv.org/) provides a graphical user interface for biologists to perform annotation and visualization with genomic data. Alternative open-source tools include SnapGene Viewer (https://www.snapgene.com/snapgene-viewer), DNAPlotter (83), Gview Server (84), PlasMapper (85), and AngularPlasmid (https://angularplasmid.vixis.com/index.php), in addition to subscription-based options such as Geneious Prime (https://www.geneious.com/). Circular plasmid sequences can also be visualized on R using the plasmapR package (https://github.com/BradyAJohnston/plasmapR). For comparative plasmid analysis, BLAST Ring Image Generator (BRIG) allows users to align and visualize shared regions between whole plasmid sequences, although it is limited to plasmids with high homology or sequence identity (48). Plasmid synteny can be visualized in linear representations using Clinker (49), Easyfig (86), GenoFig (87), or similar implementations in R (genoplotR [88], *gggenomes* [https://github.com/thackl/gggenomes]) or Python. Tools for constructing circular plots or viewing plasmid synteny or regions of recombination are also available in R (89–92) and Python (pyCircos, pyCirclize, pyGenomeViz and plotMyGBK).

### Identifying plasmid spread

Identifying similar plasmids or plasmid clusters can be challenging as plasmids within the same strain, species, or genus do not necessarily share a common “backbone.” Members of a population can exchange plasmids or undergo recombination to produce novel, hybrid plasmids. Genomic surveillance of an AMR plasmid is considered more challenging than core genome-based typing. The bacterial core and accessory genomes evolve at varying rates, making it fundamentally difficult to establish an evolutionary threshold for plasmids.

Bacterial plasmids can be grouped into distinct clusters or plasmid taxonomic units (PTUs) based on their core or “backbone” gene content, such as in pMLST (93), or by comparing SNPs between conserved regions (94, 95), shared *k*-mer content (50, 96, 97), or pairwise ANI (average nucleotide identity) (98, 99). Plasmids of the same family (e.g., same Rep type) typically share the same set of core genes, which are often essential housekeeping genes for plasmid maintenance, replication, segregation, and transfer. Plasmid clustering has been applied to infer transmission events within a single or multiple linked populations (29, 100). Transmission analysis is applied in outbreak investigation where one or multiple plasmids are suspected to confer AMR properties within a bacterial sub-population. In this case, short reads or contigs can be mapped to an “index” plasmid to predict the putative presence of a plasmid using a certain coverage and similarity threshold (101, 102). This method usually works more accurately with short-term outbreaks, where the plasmids have accumulated negligible structural changes since the index plasmid.

Often, the very first step in identifying homologous plasmids is to establish an empirical threshold to define plasmid similarity. For instance, a recent study utilizing an observational data set of clinically derived plasmids established a similarity threshold of 95% plasmid gene content at >99% nucleotide to infer horizontal transmission events (103). Despite this, the same threshold might not be accurate when the context changes, such as in long-term outbreaks. When investigating plasmid transmission, similarity threshold can vary, with some tools, such as MOB-suite (51) and PlasmidSeeker (55), utilizing a pre-set threshold, and others utilizing local benchmarked thresholds (101–107).

Popular tools for clustering plasmids are summarized in Table 2. Mash (108, 109) is an alignment-free method that clusters plasmids based on the fraction of shared *k*-mers or Jaccard index (109). Similarly, MOB-cluster (50, 51) and mge-cluster (110) group plasmids based on *k*-mer content. The former clusters plasmids based on pairwise Mash distance against a reference plasmid database, while the latter generates plasmid clusters based on presence-absence matrix of unitigs (or *k*-mers) which are then transformed into Jaccard distances. Unlike the MOB-cluster, the mge-cluster is reference-free and does not use a single pre-defined Mash threshold to cluster plasmid sequences, resulting in its wider applicability across distinct taxonomic groups. On the other hand, COPLA is alignment-based and clusters plasmids into PTUs based on pairwise ANI score against a curated plasmid database (99). Both Mash (alignment-free) and COPLA achieve good correlation when similar genomes are compared, but this correlation declines with more divergent genomes or plasmids (e.g., less than 90% ANI) (109). Despite that, both tools do not consider genome synteny or rearrangements in plasmids. A recent tool, pling (111), takes into account the structural changes (rearrangements, insertions, and deletions) when clustering plasmids and prevents over-clustering of plasmids based on promiscuous transposable elements. Pling demonstrates exceptional performance when it comes to grouping recently related plasmids with an identifiable core backbone.

**TABLE 2:** Tools for clustering plasmid and transmission analysis

Tools	Publication year	Description
Mash (109)	2016	An alignment-free method that utilizes the MinHash technique to compress large sequences to sketch representations followed by calculation of the Jaccard index (i.e., fraction of shared *k*-mers). Mash performs better with genetically similar plasmids, such as in outbreak or short-term transmission that involves little structural alterations (68, 112). Mash has been incorporated into MOB-suite, mge-cluster, and SeqSphere^+^ plasmid transmission detection pipeline. Available as a command line and desktop application. Accessible at: https://github.com/marbl/Mash
Mob-cluster (50, 51)	2018	A sub-utility of MOB-suite that performs clustering based on pairwise Mash distance (linkage clustering) against a reference plasmid database and groups plasmids into different mob-clusters. As the MOB-cluster depends on an existing database to cluster plasmids, it does not perform well on novel plasmids and is favored toward Enterobacteriaceae plasmids. Available as a command line tool. Database was last updated on 4 June 2024. Accessible at: https://github.com/phac-nml/mob-suite
COPLA (99)	2021	COPLA clusters plasmids into plasmid taxonomic units (PTUs) based on pairwise ANI score. Each plasmid is a node, and individual nodes with an ANI score >70% along 50% of the smallest plasmid length are linked by edges. A minimum of four members is needed to define a PTU (or when a query sequence cluster with at least three plasmids from the reference database). COPLA performs better with larger data sets. Available as a command line and web tool. Accessible at: https://github.com/santirdnd/COPLA (command line), https://castillo.dicom.unican.es/copla/ (web server)
mge-cluster (110)	2023	Reference-free clustering tool generates plasmid clusters based on presence-absence matrix of unitigs (*k*-mers of a fixed size) which were then embedded in a two-dimensional representation using the openTSE algorithm. Allows for customizable Mash cluster thresholds. Available as a command line tool. Accessible at: https://gitlab.com/sirarredondo/mge-cluster
SHIP (113)	2023	Identifies horizontally transmitted regions (e.g., AMR regions) in phylogenetically distant plasmids. Initial clustering of plasmids based on Jaccard similarity of gene content. Available as command line tool. Accessible at: https://github.com/AbeelLab/plasmidHGT
Pling (111)	2024	Epidemiological clustering of plasmids takes into consideration structural events, such as plasmid rearrangement and indels, while reducing over-clustering due to shared transposable elements. Available as a command line tool. Accessible at: https://github.com/iqbal-lab-org/pling
SeqSphere+ (112)	2024	Real-time plasmid transmission detection pipeline based on Mash distance. Includes plasmid reconstruction and typing using MOB-suite. Users can visualize output on the same platform. Available as a desktop application. Accessible at: https://www.ridom.de/seqsphere/u/Long-read_Data_Plasmid_Transmission_Analysis_Module.html

To visualize plasmid clusters and networks, Cytoscape (114) and igraph (68, 115) are popular options. Cytoscape is an open-source, interactive desktop application for biological network visualization. Cytoscape enables the integration of multiple biological features, including genotypes and phenotypes, to display relationships between traits and has been used to visualize plasmid networks in various studies (96, 116, 117). Other tools include web applications such as pATLAS and PLACNETw that integrate both plasmid reconstruction and visualization (57) and Visone (118).

Plasmid identification, typing, and clustering have been applied to characterize plasmids that drive the emergence of “successful” clones of multi-drug resistant (MDR) pathogens, identify high-risk plasmids, and study plasmid genomic epidemiology across geographical and ecological barriers. For instance, genomics has been used to resolve AMR plasmids and reconstruct plasmid movements and evolution (29, 74, 75, 119–121). Plasmids have also been associated with the emergence of certain drug-resistant lineages, such as the plasmid pOXA-48 carrying the carbapenemase gene *bla_OXA-48_* in *Klebsiella pneumoniae* (122, 123), the IncF plasmids carrying *bla_CTX-M_* genes in *Escherichia coli* and *K. pneumoniae* (124, 125), and the ColV plasmids in *E. coli* ST58 (126). A recent review also highlights how plasmids are important in understanding AMR dissemination across One Health sectors, highlighting examples on plasmid-mediated colistin and carbapenem resistance spread (127). Together, these examples reinforce the significance of incorporating AMR plasmids in genomic surveillance of human bacterial pathogens to inform emerging resistance and guide control strategies.

## PLASMID DATABASES

Multiple databases exist and can be used at all stages of the plasmid analyses described above, from plasmid identification (MOB-suite, PlasmidFinder), gene annotation or screening (Prokka, Abricate) to plasmid typing. These databases vary in size and diversity, with many housing a larger collection of plasmids from gram-negative species than gram-positive, leading to an underrepresentation of some pathogenic species. These sampling biases, along with many of the self-curated databases—which are often not regularly updated or maintained—remain a challenge for consistency across research and public health due to the lack of universally accepted or “gold standard” database.

In August 2014, the NCBI Plasmid Genome database recorded 4,418 complete plasmid sequences from bacteria, with the majority belonging to the phyla Proteobacteria (46.5%) and Firmicutes (24.6%) (128). This number increased drastically to 12,091 bacterial plasmids in May 2018 (50). Most plasmid databases are freely accessible and contain information on whole plasmid, gene, or protein sequences (Table 3). The National Center for Biotechnology Information (NCBI) (https://www.ncbi.nlm.nih.gov/) Nucleotide database is a centrally organized repository and houses an expansive collection of plasmid, gene, and protein sequences from diverse taxa. Although NCBI itself does not have a dedicated plasmid-only database, users may opt to download complete plasmid assemblies from a particular taxonomic group using the *ncbi-genome-download* (https://github.com/kblin/ncbi-genome-download) command line utility with customizable parameters. An alternative web-based interface for retrieving plasmid assemblies, based on known accession, is the Batch Entrez utility (https://www.ncbi.nlm.nih.gov/sites/batchentrez). The “sister” alternative to NCBI is PLSDB, which houses a collection of non-redundant plasmid sequences from NCBI with searchable metadata (38–40). PLSDB performs replicon typing by default and can be run on a web server or local computer. Another example, plasmid ATLAS (pATLAS) (http://www.patlas.site) (41) hosted on the web server allows the user to look for plasmids against the NCBI RefSeq database and visualize selected plasmid networks within and between taxa to look for putative evolutionary changes. Besides these centrally organized databases, locally curated databases have been established (67, 129), including the ones utilized by MOB-suite (50), COMPASS (54), and more recently the IMG/PR database (130) for metagenomic data.

**TABLE 3:** Plasmid databases that are openly accessible

Database	Publication year	Description
National Center for Biotechnology Information (NCBI)	N/A^*a*^	NCBI nucleotide database for general genome sequence queries and download. Users can restrict search to “Genetic compartments: Plasmid” for the desired species or genus. Command line utility (*ncbi-genome-download*) available to download sequences with customizable parameters. Accessible at: https://www.ncbi.nlm.nih.gov/nucleotide/
PLSDB (131, 132)	2019	Open access database containing plasmid sequences retrieved from NCBI Nucleotide. Sequence metadata is available for download. Users can upload their own plasmid FASTA sequence and search for similar matches using the BLAST utility. Metadata filtering is available. The database was last updated on 31 May 2024. Accessible at: https://ccb-microbe.cs.uni-saarland.de/plsdb/
pMLST (93)	2018	A database for typing plasmids of the A/C, I1, HI1, HI2, F, and N incompatibility groups, mainly found in Enterobacteriaceae family. Accessible at: https://pubmlst.org/organisms/plasmid-mlst
pATLAS (133)	2018	Web browser database with interactive visual tools to explore plasmid relationships, e.g., graph-based plasmid networks. Plasmid identification from assemblies. Plasmid sequences were retrieved from the NCBI RefSeq database. Metadata filtering is available. The database was last updated on 20 September 2018. Accessible at: http://www.patlas.site/

^*a*^ N/A, not applicable.

## IMMEDIATE CHALLENGES FOR PLASMID GENOMICS

WGS has been applied in public health to study bacterial genomes and AMR determinants. Although plasmids play a significant role in pathogen adaptation and survival, significant knowledge gaps exist within the niche of AMR plasmid genomics. The incorporation of plasmids into epidemiological surveillance workflows represents a major barrier in public health research, especially when it comes to resolving structural variations in plasmids. Nonetheless, novel *in silico* methods, algorithms, and tools have been established for plasmid typing and clustering analysis, paving a promising path for AMR plasmid surveillance. Below, we highlight some of the persisting challenges in this rapidly emerging field.

### Addressing sampling biases in plasmid databases

Sampling bias has been a chronic issue in pathogen research. Biases can manifest in the form of: (i) sequencing bias, where most isolates being sequenced were chosen on a phenotype-based selection strategy (7) or from culturable organisms; (ii) sampling bias, where most studies examined the diseased population; (iii) species bias, where the majority of sequencing data were derived from few primary pathogenic species, e.g., from the family Enterobacteriaceae; and (iv) geographical and sampling site bias, where most sequencing data were generated from countries or regions with affordable sequencing capacity and from host sites which are more accessible or prone to infections. Conventional sequencing also often misses low copy number plasmids, such as the pINV plasmids in *Shigella* spp. (134), which can harbor important genes for pathogenicity and host adaptation. Together, these sampling and sequencing biases could lead to an underrepresentation of plasmids from minority species that display close interactions with potentially pathogenic species in the same niche, e.g., the mammalian gut.

More recently, large-scale population-level plasmid surveys are moving toward a “One Health” approach, expanding from clinical human-centric surveillance to identifying geotemporal links in AMR dissemination (135, 136). Many plasmids recovered from the natural environment were novel and distinct to those characterized from human or clinical samples. Studies have found a greater association between plasmid distribution and ecology (niche or biome) than geography, suggesting limited gene flow between ecosystems (3, 20, 137, 138). For instance, Enterobacterales plasmids (68) were shared between diverse hosts (human and animal), genera, and ecological niches. Numerous studies have also found that plasmid carriage differs between closely related strains or species. For instance, different *Salmonella* serotypes were found to be highly variable in their plasmid carriage, with some plasmid lineages inherited vertically within certain non-typhoidal serovars (139, 140). Together, these findings reinforce the importance of navigating diverse host and ecological contexts in AMR plasmid studies and future cross-sectoral plasmid surveillance applications.

### Establishing taxon-specific plasmid typing schemes

Current plasmid classification schemes were established based on shared genes, such as replication and relaxase genes, but few have accounted for species-specific differences. Certain pathogenic species, such as *Escherichia coli*, have better established classification schemes and benchmarking than others (141, 142). In a study assessing taxonomy-specific plasmidome (143), global mapping of >10,000 prokaryotic plasmids across different genera divided plasmids into distinct taxonomic units that did not always correlate with other established typing schemes. Phylogenetic barriers remain a key player in shaping host-specific plasmidome by limiting inter-taxonomic plasmid exchange (143), although studies on the human microbiome have found subsets of plasmids that were widely disseminated across phylogenetically distant bacterial taxa (144). In a complex microbial community, coevolution of plasmids should be considered when investigating long-term within-host plasmid evolution (145). Building upon these findings, having a taxon-specific plasmid classification scheme with sufficient flexibility to account for inter-phylum gene exchange would present a better alternative for studying plasmids in drug-resistant pathogens.

### Dealing with linear plasmids carrying AMR genes

Linear plasmids carrying AMR genes were generally rare in pathogenic bacteria but have been gaining increasing attention in recent years. Linear plasmids are distinguished from circular plasmids by their structure (i.e., terminal ends) and replication machinery (146). Linear plasmids carrying resistance genes for critical or last-line antimicrobials have been identified from Enterobacteriaceae (94) and *Enterococcus* spp. (*E. faecium* and more recently *E. faecalis*) (147, 148). Interestingly, several studies have discovered evidence of conserved linear plasmid “lineages” in *E. faecium* (149) and *Micrococcus* (150), suggesting that many of these plasmids were in fact inherited and maintained under strong vertical selective pressures and fitness costs.

Bioinformatic methods to detect linear plasmids from hybrid assemblies have been applied to study linear plasmids in Enterobacteriaceae (94), presenting an alternative approach to traditional methods using pulsed-field gel electrophoresis (PFGE) with nuclease treatment (151). The identification and typing of linear plasmids remain a challenge in public health, as some of these plasmids do not carry the typical replicon types found in circular plasmids (149). Similarly, it is often difficult to distinguish between “true” linear plasmid, linearized circular plasmids, and fragmented chromosomal contigs in the absence of long reads. Assemblers also sometimes struggle with assembling linear plasmids due to the difficulty in resolving the terminal inverted repeats on both ends of these plasmids (44).

## CAN LONG-READ SEQUENCING SOLVE ALL OUR PROBLEMS?

The recent advances in Oxford Nanopore long-read sequencing have made it possible to reconstruct complete bacterial genome assemblies (152), with the option for automated assembly pipelines (153). The availability of long-read tools and reduced sequencing costs would significantly expand the current number of complete bacterial genome and plasmid sequences on public repositories. Several key areas would receive benefit from this advancement, including the study of plasmid evolution within host or across taxonomy, and the implementation of plasmid genomics in AMR surveillance. The ability of long reads to resolve structural variants and long repetitive elements in plasmids would mean a step further in understanding plasmid epidemiology. Reconstructing large, multi-AMR regions on plasmids allows us to understand the vertical and horizontal transmission dynamics of these genes in different bacterial communities. With these advancements, there is also a growing need for higher throughput bioinformatic tools and workflows to manage the increasing demands of sequence processing and analysis.

## CONCLUSION

AMR continues to play a central role in public health surveillance. Understanding the genomic and physiological properties of AMR plasmids is crucial for effective drug-resistant pathogen surveillance. Although *in silico* technologies offer an attractive approach to traditional typing in AMR plasmid research, experimental data remain crucial to elucidating the biology of these elements and the *in vivo* interactions of plasmids within microbial communities. As novel tools continue to emerge in this growing field, plasmid genomics will play a pivotal role in shaping future AMR surveillance framework and addressing gaps in pathogen evolution.
